# In-hospital mortality as an outcome indicator for air pollution health risk assessment: data utility and research challenges

**DOI:** 10.3389/fpubh.2026.1628326

**Published:** 2026-02-04

**Authors:** Yakun Zhao, Yuansong Zhuang, Shiyu Zhang, Zhongjie Fan

**Affiliations:** 1 Department of Cardiology, Peking Union Medical College Hospital, Peking Union Medical College and Chinese Academy of Medical Sciences, Beijing, China; 2 Department of Internal Medicine, Peking Union Medical College Hospital, Peking Union Medical College and Chinese Academy of Medical Sciences, Beijing, China; 3 Department of Dermatology, State Key Laboratory of Complex Severe and Rare Diseases, Peking Union Medical College Hospital, Chinese Academy of Medical Sciences and Peking Union Medical College, Beijing, China

**Keywords:** air pollution, data mining, in-hospital mortality, outcome indicator, risk assessment

## 1 Introduction

Numerous studies have delved into the adverse impact of air pollution on health, such as increased hospital visit risks of cardiovascular diseases and respiratory diseases, and identified the association between short- and long-term exposure to ambient air pollution and increased risks of developing and dying from diseases, including cardiovascular diseases, respiratory diseases and so on (1–3). The global exposure to this risk is rapidly intensifying. The Global Burden of Disease Study 2019 identified ambient particulate matter pollution as one of the risk factors with the largest increases in exposure from 2010 to 2019, and ambient air pollution led to approximately 6.67 million deaths globally in 2019 (4). We have noticed that recently an increasing number of studies have focused on a health outcome—in-hospital mortality. Therefore, to ground our discussion, we reviewed articles published in English over the last 10 years that focus on this specific outcome (Table 1) (5–18). This viewpoint aims to highlight the significance of in-hospital mortality as an outcome indicator and the potential of utilizing hospitalization records to advance our understanding of the air pollution-health linkage.

**Table 1:** Characteristics of the studies involving ambient air pollution and the risk of in-hospital mortality.

**References**	**Country**	**Pollutants**	**Exposure**	**Outcomes**	**Study design**	**Controlled factor**	**Conclusion**
Argacha et al. (5)	Belgium	PM_2.5_, PM_10_, NO_2_, O_3_	Short-term, according to residential address	In-hospital mortality in STEMI population^*^	Case-crossover	Temperature	No association was observed.
Im et al. (6)	Korea	PM_10_, SO_2_, CO, O_3_	Short-term, according to residential postcode	Ninety-day in-hospital mortality in critically ill patients	Cox regression	Demographic factors, socioeconomic status, type of ICU, comorbidities	Short-term exposure to CO and O_3_ were associated with higher pulmonary disease-related 90-day mortality in patients with COPD.
Barret et al. (7)	USA	PM_2.5_, O_3_	Long-term, according to hospital address	In-hospital mortality in patients with sepsis	Logistic regression	Demographic factors, socioeconomic status, treatment, comorbidities	Long-term exposure to O_3_ was associated with higher mortality in patients with sepsis.
Dominguez-Rodriguez et al. (8)	Dust Belt-Canary Islands, Spain	Saharan dust events (PM_10_ >50 μg/m^3^)	Short-term, monitoring stations measurement and dust modeling	Thirty-day in-hospital mortality in patients with HF	Logistic regression	Demographic factors, comorbidities, lab test, treatment, etc.	Saharan dust events with PM_10_ >50 μg/m^3^ were associated with increased risk of in-hospital mortality in patients with heart failure.
White et al. (9)	Dublin, Ireland	PM_10_, SO_2_	Short-term, average values of monitoring stations	Thirty-day in-hospital mortality in ED patients	Logistic regression	Comorbidity score	Short-term exposure to PM_10_ and SO_2_ was associated with higher in-hospital mortality.
Desperak et al. (10)	Upper Silesia and Zaglebie Metropolis	PM_10_, SO_2_, NO, NO_2_, O_3_	Short-term, average values of monitoring stations	Thirty-day in-hospital mortality in ACS and CCS patients with PCI	Cox regression	Demographic factors, comorbidities, smoking, lab test, etc.	Short-term exposure to exceeding the 3rd quartile of PM_10_ and SO_2_ were associated with increased risk of in-hospital 30-day mortality in ACS patients.
Huang et al. (11)	Kaohsiung, Taiwan, China	PM_2.5_, PM_10_, NO_2_, O_3_	Short-term, according to residential address	In-hospital mortality in STEMI patients	Logistic regression	Demographic factors, triage status, comorbidities	Short-term exposure to NO_2_ in warm season and PM_10_ in cold season was associated with higher in-hospital mortality in STEMI patients.
Keller et al. (12)	Germany	PM_2.5_, PM_10_, SO_2_, NO, NO_2_, O_3_, benzene	Long- term, according to residential address	In-hospital mortality in ischemic stroke patients	Logistic regression	Demographic factors, socioeconomic status, comorbidities, treatment, etc.	Long-term exposure to PM_2.5_, NO, SO_2_, O_3_, and benzene were associated with increased in-hospital mortality in stroke patients.
Sánchez-de Pradae et al. (13)	Spain	PM_2.5_, PM_10_, SO_2_, NO, NO_2_, CO, O_3_	Short-term, according to residential postcode	In-hospital mortality among COVID-19 population	Time-series	Meteorological factors, DOW.	Short-term exposure to PM_10_, NO_2_, and SO_2_ were associated with increased risk of in-hospital mortality due to COVID-19.
Cai et al. (14)	Four provinces, China	PM_1_, PM_2, 5_, PM_10_	Short- and long- term, according to residential address	In-hospital mortality in stroke patients	Logistic regression^†^	Demographic factors, socioeconomic status, meteorological factors, comorbidities, etc.	Short- and long-term exposure to PM_1_, PM_2.5_ and PM_10_ were significantly associated with higher risk of in-hospital mortality in stroke patients.
Cai et al. (15)	Same as above	PM_2.5_ and its components	Same as above	In-hospital mortality in stroke patients	Same as above	Same as above	Long-term exposure to PM_2.5_ and specific PM_2.5_ components were associated with higher risk of in-hospital mortality in stroke patients.
Lin et al. (16)	Same as above	PM_1_, PM_2, 5_, PM_10_	Same as above	In-hospital mortality among AMI patients	Same as above	Same as above	Short- and long-term exposure to PM_1_, PM_2.5_ and PM_10_ were significantly associated with higher risk of in-hospital mortality in AMI patients.
Lin et al. (17)	Same as above	PM_2.5_ and its components	Same as above	In-hospital mortality in AMI patients	Same as above	Same as above	Short- and long-term exposure to PM_2.5_ and its components were significantly associated with higher risk of in-hospital mortality in AMI patients.
Lai et al. (18)	Shanxi, China	PM_2.5_ and its components	Short-term, according to residential address	In-hospital mortality in HF patients	Logistic regression	Demographic factors, socioeconomic status, meteorological factors, comorbidities, etc.	Short-term exposure to PM_2.5_ and its components were associated with increased risk of in-hospital mortality in HF patients.

^*^In subgroup analysis.

^†^Cox regression for sensitivity analysis.

PM_2.5_, particulate matter ≤ 2.5 μm in diameter; PM_10_, particulate matter ≤ 10 μm in diameter; SO_2_, sulfur dioxide; NO, nitrogen oxide; NO_2_, nitrogen dioxide; CO, carbon monoxide; O_3_, ozone; ICU, intensive care unit; HF, heart failure; COPD, chronic obstructive pulmonary disease; ED, emergency department; ACS, acute coronary syndrome; CCS, chronic coronary syndrome; PCI, percutaneous coronary intervention; STEMI, ST-elevation myocardial infarction; DOW, day of week.

## 2 Value of in-hospital mortality as an outcome indicator

In-hospital mortality directly reflects the short-term and severe health deterioration caused by air pollution exposure among patients, serving as a critical health outcome indicator. Although in-hospital mortality account for only a subset of total mortality, evidence indicates consistent trends in pollutant effects between in- and out-of-hospital mortality. For instance, a Chinese study found that despite the number of out-of-hospital ischemic heart disease (IHD) deaths exceeded in-hospital deaths several times, fine particulate matter (PM_2.5_) was significantly associated with an increased risk of both in- and out-of-hospital IHD mortality (19). A study analyzing daily mortality data from 1989 to 2000 in U.S. found that elevated particulate matter concentrations were significantly associated with increased risks of both in- and out-of-hospital all-cause mortality (20). Similarly, an Italian multi-city study revealed that PM_10_ increases significantly linked to higher alls-cause mortality risks, with no significant difference in effects on in- and out-of-hospital mortality (between-group comparison *P* = 0.817) (21). These findings collectively support the validity of in-hospital mortality as an outcome indicator for assessing the mortality risks due to ambient air pollution.

Nevertheless, from the perspective of the complexity of causal association analysis, in-hospital mortality has unique research value compared to out-of-hospital mortality. First, deaths occurring in hospitals benefit from more comprehensive clinical evaluations that minimize misclassification of death causes (22). In contrast, out-of-hospital deaths are prone to classification errors in cause of death and frequently require autopsies to verify causes (23, 24). Second, determining causality in out-of-hospital deaths is complex due to influencing variables including the quality of pre-hospital emergency care (25) and latent disease progression (26). These critical confounders are frequently undocumented in mortality records, leading to underreporting of true contributing factors (27). However, the information on important risk factors for death (such as the baseline health status and treatment) of hospitalized patients are more comprehensive, which helps reduce confounding factors during the analysis and form more rigorous causal inferences. Finally, compared with out-of-hospital deaths (even down to the minute), which supports the construction of hourly exposure lag models and is beneficial for identifying the critical exposure period and strengthening causal inference.

## 3 Advantages of utilizing hospitalization records

Most studies in Table 1 utilize existing hospitalization records to evaluate the relationship between air pollution exposure and in-hospital mortality risk. This approach represents a valuable form of medical data mining, transforming routine clinical data into valuable research findings. Using existing hospitalization records to analyze the impact of air pollution on in-hospital mortality offers the following advantages:

First, standardized hospitalization records ensure data consistency. Taking China's hospitalization record system as an example, the front page of medical records adopts structured data formats, offering information on patient demographics, comorbidities and complication diagnoses, in-hospital mortality outcomes, surgeries, invasive treatments, and so on. For nationwide hospitalization registration information, standardized medical records and quality control procedures related to medical insurance provide highly consistent and well quality-controlled data for conducting large-scale, multi-center investigations.

Second, hospitalization records provide more critical clinical information than mortality registration systems. Geographic information in hospitalization records (e.g., patient residential addresses) can be used to correlate high-resolution air pollution monitoring data for more precise exposure assessment. These extensive clinical data enable researchers to: (1) explore susceptible subgroups through stratified analysis (e.g., patients of different age groups or with varying comorbidities); (2) examine the influence of socioeconomic factors (marital status, occupation, etc.).

Most importantly, these studies can be conducted without extra recruitment or sample collection costs. By analyzing existing clinical data, studies can provide substantial evidence for public health decision, such as identifying the benefits of air pollution control and vulnerable populations requiring more protection.

## 4 Challenge of air pollution related in-hospital mortality study

Despite the aforementioned advantages, there are some issues that need further attention when utilizing hospitalization data for air pollution related in-hospital mortality research.

First is the potential exposure bias. Current studies usually use pre-admission exposure (e.g., 1 week before admission) as individual exposure, which did not assess in-hospital exposure effects. This may introduce exposure bias, particularly for patients with long hospital stays. We recommend routinely restricting in-hospital death windows (e.g., within 30 days) as sensitivity analysis to check result robustness. Future studies should take indoor air pollution during hospitalization by indirectly estimating indoor air pollution based on indoor-outdoor correlation coefficients from prior studies (28, 29), or directly measuring indoor pollution levels. Relying on monitoring station data to assess exposure may inaccurately reflect individual exposure due to spatial heterogeneity. We suggest integrating exposure by using high-spatial-resolution pollution measurement methods [e.g., combined satellite retrievals and ground-based measurements (30)] based on patients' address information to improve exposure assessment precision.

Second, selection bias must be considered. Rapid-onset diseases may cause death before hospital admission. In some regions, cultural preferences may lead to a higher likelihood of home deaths near the end of life (31). These factors can result in an underestimation of in-hospital mortality burden associated with air pollution, as demonstrated by discrepancies between in-hospital mortality data and population-level mortality data (32–34). We suggest combining hospitalization records with death registry data to analyze both in-hospital and peri-hospitalization deaths. This would help assess the impact of unrecorded peri-hospitalization deaths and provide valuable insights for future research. Additionally, cross-regional studies can help minimize the influence of cultural differences on the results.

Finally, confounding control and generalizability need improvement. As shown in Table 1, most existing studies use logistic regression models at the individual level to estimate air pollution related in-hospital mortality. However, lifestyle factors (e.g., smoking status) and genetic backgrounds are often unavailable in hospitalization records, potentially introducing unmeasured confounding in logistic regression. We recommend adopting case-crossover designs to control unmeasured time-invariant confounders through self-matching (35, 36). And the results from case-crossover studies approximate relative risks (35) and thus offer greater generalizability than odds ratios from logistic regression. Furthermore, multicenter studies can further enhance the generalizability of the findings by reducing regional heterogeneity.

## 5 Conclusions

Investigating in-hospital mortality as an outcome indicator for air pollution health effects complements current air pollution health studies. Utilizing hospitalization records for such research is a feasible and economical method. We recommend maximizing the utility of available medical records to conduct multicenter, high-quality studies, offering scientific evidence for public health policy against pollution-related health risks.
